# Degree of Satisfaction With Telemedicine Service Among Health Practitioners and Patients in Saudi Arabia

**DOI:** 10.1155/ijta/5527659

**Published:** 2025-03-07

**Authors:** Salem S. Albagmi, Mohammed S. Shawaheen, Humood Albugami, Albandari Alshehri, Raghad Alblwie, Wadha Alsiari

**Affiliations:** ^1^Health Information Management Department, Prince Sultan Military College of Health Sciences, Dhahran, Saudi Arabia; ^2^Public Health Department, King Faisal University, Al Ahsa, Saudi Arabia; ^3^Continuous Quality Improvement and Patient Safety Department, King Salman Armed Forces Hospital, Tabuk, Saudi Arabia

## Abstract

The satisfaction level of patients and physicians with telemedicine services should be comparable to on-site visits if this technology is to be widely adopted. The degree of satisfaction can be used to assess the performance of any healthcare service. Therefore, in this cross-sectional study, we used a five-point Likert scale to evaluate the level of satisfaction with telemedicine by administering survey questionnaires to both patients and physicians online residing across Saudi Arabia. The questionnaires were divided into three parts: consent and summary, demographics, and the survey questions to determine satisfaction level with telemedicine. All statistical analysis was performed using SPSS Version 27. The results showed high satisfaction levels with telemedicine services by both patients and physicians where the age and gender of participants were influential in determining this outcome. Residential areas affected satisfaction levels with telemedicine in physicians but not in patients. Previous experiences with these services influenced the satisfaction of patients with those having previous experience reporting higher satisfaction. However, when the degree of satisfaction between patients and physicians was compared, the results were insignificant showing that the overall perception towards telemedicine usage is similar between healthcare providers and receivers. To conclude, our study showed that the experiences of patients and physicians with telemedicine services are satisfactory; however, certain areas regarding telemedicine usage warrant further improvement.

## 1. Introduction

Telemedicine is defined as the application of information technology to facilitate the transmission of medical information and aid in communication across long distances between different locations [1]. There is exponential growth in the telemedicine market globally and is projected to expand further in the coming 5 years [2]. One reason for this increased usage of telemedicine is the recent pandemic of COVID-19 in 2020 [3] which also influenced healthcare delivery in a variety of ways. In-person visits were largely replaced with telemedicine services for the delivery of healthcare during pandemic. However, many still argue the insufficiency of virtual care when compared with face-to-face visits in managing ailments [4].

The main factors that govern the acceptance and usage of telemedicine among physicians include facilitating conditions, attitude to use, self-efficacy, compatibility, subjective norms, ease of use, perceived usefulness, and behavioral control [5]. Other concerns shared by both patients and clinicians include the inability to perform physical examinations, difficulty in ordering the required tests, and the evaluation of mental health. Concerns for the privacy of the information shared via virtual consultation are also an issue [6]. Therefore, for telemedicine to be widely adopted, both patients and physicians should be satisfied with it as much as they are with on-site consultations [7]. Satisfaction is identified as one of the indicators to evaluate the performance of a healthcare service [8]. It mirrors the expectations and values of patients concerning the numerous aspects of healthcare services. When the care expected and received is aligned, patients appear to be satisfied [9].

A majority of surveys have been conducted to assess the level of satisfaction of both clinicians and patients with telemedicine considering numerous factors in many countries including Saudi Arabia (SA). However, most of the studies undertaken in SA focused on determining the performance, satisfaction, and efficiency of telemedicine during the COVID-19 pandemic where it was necessary to provide healthcare remotely to prevent the spread of infection. Therefore, satisfaction with healthcare could have been influenced by the limited resources at disposal for the provision of care. Only a limited number of studies thus far have focused on whether the trend of the degree of satisfaction remains consistent in the postpandemic era in SA. Therefore, the central aim of this study is to assess the level of satisfaction with telemedicine services in health providers and receivers where on-site visits are as much a possibility as telemedicine services.

## 2. Materials and Methods

### 2.1. Study Design and Population

This is a cross-sectional study consisting of 248 physicians and 586 patients from different demographic locations across SA. The inclusion criteria are as follows: both genders, above 18 years of age, and have used telemedicine services before for any reason.

The sample size was determined using the following formula:

 $$\begin{array}{c} n=\left(Z\frac{\alpha }{2}+Z\;\beta \right)\;2*\frac{p\;1\left(1-p\;1\right)+p\;2\;\left(1-p\;2\right)}{\left(p\;1-p\;2\right)}2 \end{array}$$

where *Z* *α*/2 is the critical value of the normal distribution at *α*/2 (e.g., for a confidence level of 95%), *α* is 0.05 and the critical value is 1.96), *Z* *β* is the critical value of the normal distribution at *β* (e.g., for a power of 80%, *β* is 0.2 and the critical value is 0.84), and *p*1 and *p*2 are the expected sample proportions of the two groups. The sample size was determined to be 194 for patients and physicians, respectively. The survey was administered to 700 patients and 300 physicians; however, only 586 patients and 248 physicians responded, resulting in a response rate of 302% and 128%, respectively.

### 2.2. Ethical Consideration

This study was approved by the Institutional Review Board (IRB) of Prince Sultan Military College of Health Sciences (IRB# IRB-2023-HIM-046). The participation in survey was voluntary. Confidentiality and privacy of participants were maintained throughout the entirety of the study. The data was stored in password-secured personal computers to which only a few people concerned with the study had access.

### 2.3. Data Collection

The survey was made using Google Forms and administered to the desired population via email. For patients, the form consisted of three sections with a total of 28 questions. The first part of the questionnaire contained a summary of the questionnaire and consent to participate. The second part of the questionnaire consisted of four statements that presented demographic characteristics. The third and final section contained 23 questions that assessed patient's satisfaction with the telemedicine service. For physicians, the form also consisted of three sections with a total of 12 questions. The first part of the questionnaire contained a summary of the questionnaire and consent to participate. The second part of the questionnaire contained six statements that presented demographic characteristics. The third and final section contained five questions to measure doctors' satisfaction with the telemedicine service. The Likert scale comprised of five options ranging from *very satisfied* to *very unsatisfied* was used to assess the third part of the surveys for both the patient and physician.

### 2.4. Data Analysis

The data of the study was analyzed using IBM's Statistical Package for Social Sciences (SPSS) Version 27. Percentage and frequency distributions of all categorical variables were calculated to determine how many responses were for each category. The normality of the gathered data was determined using the Shapiro–Wilk Test. A chi-square test was carried out to evaluate whether the differences between the level of satisfaction of physicians and patients (dependent variables) and demographics (independent variables) were significant. Wilcoxon test was used to compare if there was a significant difference between satisfaction levels with telemedicine of physicians and patients. A difference of ≤ 0.05 was considered significant.

## 3. Results and Discussion

This study analyzed questionnaires from 248 physicians and 586 patients. A greater percentage of physicians was male (56.5%) compared to females (43.5%), whereas in patients, the percentage of females (54.9%) was higher than that of males (45.1%). The majority of the patients (33.8%) and physicians (43.5%) were between the ages of 40 and 49 years. Most physicians were Saudi nationals (79.8%) residing in the eastern region (45.2%) of SA. In contrast, a vast majority of patients were from the northern region (35.2%). 90.3% of physicians confirmed having provided telemedicine services before, whereas only 50.9% of the patients said that they have availed telemedicine services previously (Tables 1 and 2).

Among physicians, 37.9% and 46.8% were satisfied and very satisfied with telemedicine, respectively. 12.9% of the physicians were neutral, and only 2.4% were dissatisfied. Surprisingly, none of them were very dissatisfied. The main reason for the high overall satisfaction with telemedicine can be attributed to the fact that the majority of the physicians were satisfied with how they catered to the needs of the patients and provided the emotional support and relevant information needed. They were also satisfied with how actively the patient participated in their interaction (Table 3). In another study, similar results have been reported, where 68.9% of the healthcare providers showed a positive attitude towards using and adapting telemedicine services [10]. Gillman-Wells, Sankar, and Vadodaria [11] also showed that 70% of plastic surgeons are willing to accept telemedicine services as part of their practices. Openness to telemedicine as evidenced by numerous studies can also be motivated by the personal advantages it can provide to physicians such as flexible and convenient schedules or nonpersonal benefits such as improving the quality of healthcare for patients.

As with physicians, patients also appeared to be highly satisfied with telemedicine. Among them, 42.3% and 33.1% were very satisfied and satisfied, respectively, whereas only 3.8% and 1.7% were dissatisfied and very dissatisfied, respectively. 19.1% of patients were neutral; they were neither satisfied nor dissatisfied with telemedicine. The high satisfaction of patients with telemedicine can be owed to the high quality they encountered or expected in terms of the displayed image, audio, emotional support, comfort, scheduling, consistency, communication, understanding, and recommendations by the physician as shown in Table 4. This also resulted in patients preferring virtual care over on-site visits since it is more cost-effective. The majority also agreed to use telemedicine again in the future. The high degree of patient satisfaction with telemedicine has been reported in other studies as well undertaken by Poulsen et al. [12] and Abdulwahab and Zedan [13]. Telemedicine also reduces the need to travel, which means it can make healthcare accessible in areas where it is generally limited and to people who cannot travel due to mobility issues making it a feasible option for the provision of healthcare in the near future.

Table 5 assesses physician satisfaction with telemedicine services, examining various demographic factors in the postpandemic era in SA. It reveals that satisfaction levels vary significantly across gender and age groups, with *p* values of 0.044 and 0.038, respectively, indicating that these factors influence physician satisfaction. Males reported higher levels of neutrality and dissatisfaction compared to females, while older age groups (40–49 and 50–59) tended to report higher satisfaction than younger ones. Nationality did not significantly affect satisfaction (*p* value 0.275), suggesting similar satisfaction levels among Saudi and non-Saudi physicians. The residential area showed a highly significant difference (*p* value < 0.001), with the Eastern region reporting the highest levels of satisfaction. Prior experience with telemedicine did not significantly impact satisfaction (*p* value 0.056), indicating that both experienced and inexperienced physicians reported similar levels of satisfaction. These findings highlight specific demographic influences on physician satisfaction with telemedicine, offering insights for improving telemedicine services in the postpandemic landscape. A study by Aldakhil, Alharbi, and Alomair [14] showed contrasting results where age and gender did not influence the satisfaction of physicians with telemedicine. This difference could be because of the limited sample size in both the studies which might introduce some inaccuracy in analyzing the results.

Table 6 evaluates patient satisfaction with telemedicine services in the postpandemic era in SA. Significant differences in satisfaction levels are observed across gender, age, and prior telemedicine experience. Females report higher satisfaction than males, as indicated by a *p* value of 0.011. Age-related differences are also significant (*p* value < 0.001), with younger patients (18–19 years) generally more satisfied than older patients. Prior experience with telemedicine significantly influences satisfaction (*p* value < 0.001), with those having previous experience reporting higher satisfaction. Although residential area shows no statistically significant difference (*p* value 0.071), notable variations exist, particularly with higher satisfaction in the northern and southern regions. These insights suggest that while telemedicine satisfaction is generally high, targeted strategies may be needed to address specific demographic groups to maintain and improve satisfaction levels postpandemic. These results are opposite to those observed by Aldakhil, Alharbi, and Alomair [14], where age and gender did have the potential to influence satisfaction levels with telemedicine services. These contradictory results can be attributed to the difference in sample size and the demographic composition between the two studies.

**Figure 1:**
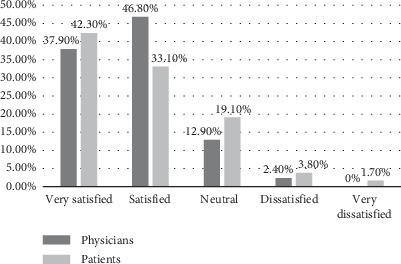
Comparison of satisfaction level with telemedicine between physicians and patients.

Figure 1 shows the distribution of satisfaction levels among both physicians and patients regarding telemedicine. The majority of physicians (37.90%) reported being very satisfied, followed closely by 46.80% who expressed being satisfied. Conversely, patients exhibited slightly higher levels of satisfaction, with 42.30% indicating they were very satisfied and 33.10% reporting they were satisfied. Dissatisfaction levels were relatively low in both groups, with only 2.40% of physicians and 3.80% of patients expressing dissatisfaction. Remarkably, no physicians reported being very dissatisfied, whereas a small percentage of patients (1.70%) fell into this category.  Table 7 provides a comparison of satisfaction levels between physicians and patients regarding using telemedicine services. Among the 248 physicians surveyed, the median satisfaction level was 2.00, indicating that half of the physicians rated the variable at or below this level. Similarly, among the 586 patients surveyed, the median satisfaction level was also 2.00. The “negative rank” and “positive rank” statistics suggest that the distribution of ranks, possibly related to satisfaction levels, was higher for patients (90.18) compared to physicians (74.63), indicating potentially higher overall satisfaction among patients. However, there was no statistically significant difference in satisfaction levels between physicians and patients regarding telemedicine services (*p* value 0.217). These insignificant differences demonstrate that satisfaction levels are likely similar in the general population irrespective of whether they are the providers or receivers of telemedicine services.

The present study leaves room for improvement. The sample size can be increased to improve the accuracy and validity of results. The survey questionnaire can be improved by using more specific, detailed, and situation-oriented questions since the present questionnaire might have introduced some biases in responses by the physicians as it was indirectly related to their competence. The education level of patients can also be used as a variable to see how it influences perceptions regarding telemedicine. Healthcare providers can be divided into subgroups in terms of their affiliation with the level of healthcare: primary, secondary, and tertiary, to demonstrate the comfort level with telemedicine services for different aspects of healthcare delivery.

## 4. Conclusion

The majority of both the patients and physicians appeared to be satisfied with telemedicine services, the level of which was found to be influenced by demographic characteristics such as age and gender. Physician satisfaction was also influenced by the area of residence offering insights for improving telemedicine services in the regions with low preference for telemedicine. Unlike physicians, perceptions towards telemedicine are influenced by prior experience with telemedicine in patients. However, when the degree of satisfaction between patients and physicians was compared, the results were insignificant showing that the overall perception towards telemedicine usage is similar between healthcare providers and receivers. This study also demonstrates the need to further improve telemedicine services in certain residential areas with low satisfaction levels so that everyone can have equitable access to the healthcare they need.

## Figures and Tables

**Table 1:** Demographics of surveyed physicians.

**Item**	**Measure**	**n**	**%**
Gender	Male	140	56.5
	Female	108	43.5

Age	25–29	22	8.9
	30–39	58	23.4
	40–49	108	43.5
	50–59	53	21.4
	≥ 60	7	2.8

Nationality	Saudi	198	79.8
	Non-Saudi	50	20.2

Residential area in Saudi Arabia	Central	46	18.5
	Eastern	112	45.2
	Northern	42	16.9
	Southern	18	7.3
	Western	30	12.1

Have you provided telemedicine service before?	Yes	224	90.3
	No	24	9.7

**Table 2:** Demographics of surveyed patients.

**Item**	**Measure**	**n**	**%**
Gender	Male	264	45.1
	Female	322	54.9

Age	18–19	104	17.7
	30–39	86	14.7
	40–49	198	33.8
	50–59	159	27.1
	≥ 60	39	6.7

Residential area in Saudi Arabia	Central	82	14.0
	Eastern	122	20.8
	Northern	206	35.2
	Southern	136	23.2
	Western	40	6.8
Have you been provided telemedicine service before?	Yes	298	50.9
	No	288	49.1

**Table 3:** Degree of satisfaction of physicians with telemedicine.

	**Very satisfied**		**Satisfied**		**Neutral**		**Dissatisfied**		**Very dissatisfied**	
	**n**	**%**	**n**	**%**	**n**	**%**	**n**	**%**	**n**	**%**
How well did you address the needs of this patient?	72	29.0	154	62.1	22	8.9	0	0.0	0	0.0
How actively was this patient involved in talking and participating in the interaction?	54	21.8	140	56.5	54	21.8	0	0.0	0	0.0
How satisfied are you with the adequacy of the information you gave to this patient?	62	25.0	158	63.7	26	10.5	2	0.8	0	0.0
How satisfied are you with the emotional support you gave to this patient?	68	27.4	148	59.7	28	11.3	4	1.6	0	0.0
Overall, how satisfied are you with the interaction?	94	37.9	116	46.8	32	12.9	6	2.4	0	0.0

**Table 4:** Degree of satisfaction of patients with telemedicine.

	**Very satisfied**		**Satisfied**		**Neutral**		**Dissatisfied**		**Very dissatisfied**	
	**n**	**%**	**n**	**%**	**n**	**%**	**n**	**%**	**n**	**%**
How easy is it to register/schedule for appointments?	218	37.2	210	35.8	142	24.2	16	2.7	0	0.0
Is the quality of the displayed visual image clear?	206	35.2	210	35.8	156	26.6	10	1.7	4	0.7
Is the audio quality clear?	238	40.6	212	36.2	122	20.8	14	2.4	0	0.0
I was able to speak freely via telemedicine.	232	39.6	214	36.5	126	21.5	10	1.7	4	0.7
I was able to understand recommendations or diagnoses.	244	41.6	194	33.1	128	21.8	18	3.1	2	0.3
The location where I received my care was comfortable.	212	36.2	228	38.9	128	21.8	16	2.7	2	0.3
The overall quality of care provided is satisfactory.	220	37.5	210	35.8	132	22.5	22	3.8	2	0.3
What do you think of the public consultation experience via telemedicine?	258	44.0	196	33.4	106	18.1	20	3.4	6	1.0
I can easily talk to my healthcare provider.	254	43.3	186	31.7	128	21.8	14	2.4	4	0.7
I can hear my healthcare provider clearly.	246	42.0	208	35.5	112	19.1	14	2.4	6	1.0
My healthcare provider is able to understand my health condition.	220	37.5	214	36.5	130	22.2	16	2.7	6	1.0
I can see my healthcare provider as if we met in person.	202	34.5	164	28.0	192	32.8	16	2.7	12	2.0
I do not need assistance while using the system.	246	42.0	166	28.3	158	27.0	14	2.4	2	0.3
I feel comfortable communicating with my healthcare provider.	236	40.3	214	36.5	116	19.8	16	2.7	4	0.7
I think the healthcare provided via telemedicine is consistent.	242	41.3	186	31.7	132	22.5	20	3.4	6	1.0
I obtain better access to healthcare services by use of telemedicine.	222	37.9	188	32.1	148	25.3	22	3.8	6	1.0
Telemedicine saves me time traveling to a hospital or a specialist clinic.	294	50.2	162	27.6	114	19.5	12	2.0	4	0.7
I do not get enough attention.	148	25.3	120	20.5	182	31.1	84	14.3	52	8.9
Telemedicine provides for my healthcare need.	224	38.2	194	33.1	144	24.6	18	3.1	6	1.0
I find telemedicine an acceptable way to receive healthcare services.	238	40.6	212	36.2	114	19.5	14	2.4	8	1.4
I will use telemedicine services again.	254	43.3	186	31.7	122	20.8	12	2.0	12	2.0
Overall, I am satisfied with the quality of service being provided via telemedicine.	248	42.3	194	33.1	112	19.1	22	3.8	10	1.7
How satisfied are you with the emotional support you received from the doctor?	230	39.2	182	31.1	148	25.3	22	3.8	4	0.7

**Table 5:** The relation between demographics and satisfaction levels of physicians.

		**Physician satisfaction**				
		**Very satisfied**	**Satisfied**	**Neutral**	**Dissatisfied**	**Very dissatisfied**
Gender	Male	56	58	24	2	0
	Female	38	58	8	4	0
	0.044					

Age	25–29	14	6	2	0	0
	30–39	24	26	4	4	0
	40–49	30	58	18	2	0
	50–59	22	23	8	0	0
	≥ 60	4	3	0	0	0
	0.038					

Nationality	Saudi	76	88	28	6	0
	Non-Saudi	18	28	4	0	0
	0.275					

Residential area in Saudi Arabia	Central	12	26	4	4	0
	Eastern	54	46	10	2	0
	Northern	20	14	8	0	0
	Southern	2	10	6	0	0
	Western	6	20	4	0	0
	< 0.001					

Have you provided telemedicine service before?	Yes	90	102	26	6	0
	No	4	14	6	0	0
	0.056					

**Table 6:** The relation between demographics and satisfaction levels of patients.

		**Patient satisfaction**				
		**Very satisfied**	**Satisfied**	**Neutral**	**Dissatisfied**	**Very dissatisfied**
Gender	Male	98	84	64	14	4
	Female	150	110	48	8	6
	0.011					

Age	18–19	70	24	8	2	0
	30–39	38	32	14	2	0
	40–49	74	74	40	6	4
	50–59	54	53	38	8	6
	≥ 60	12	11	12	4	0
	< 0.001					

Residential area in Saudi Arabia	Central	34	28	12	6	2
	Eastern	38	42	30	8	4
	Northern	90	74	34	4	4
	Southern	66	38	28	4	0
	Western	20	12	8	0	0
	0.071					

Have you been provided telemedicine service before?	Yes	152	102	26	12	6
	No	96	92	86	10	4
	< 0.001					

**Table 7:** Wilcoxon test results comparing satisfaction level with telemedicine between physicians and patients.

**Variable**	**Physicians**	**Patients**
*n*	248	586
Median	2.00	2.00
Negative rank	74.63	
Positive rank	90.18	
Asymptotic Sig. (two tailed)	0.217	

## Data Availability

The datasets used and analyzed during the current study are available from the corresponding author on reasonable request.
